# Increased Odds of Metabolic Dysfunction-Associated Steatotic Liver Disease Are Linked to Reduced n-6, but Not n-3 Polyunsaturated Fatty Acids in Plasma

**DOI:** 10.3390/biom14080902

**Published:** 2024-07-25

**Authors:** Irena Frankovic, Ivana Djuricic, Ana Ninic, Jelena Vekic, Tara Vorkapic, Sanja Erceg, Tamara Gojkovic, Ratko Tomasevic, Milica Mamic, Milos Mitrovic, Aleksandra Zeljkovic

**Affiliations:** 1 Department of Medical Biochemistry, Faculty of Pharmacy, University of Belgrade, 11000 Belgrade, Serbia; irenafrankovic@yahoo.com (I.F.); ivana.djuricic@pharmacy.bg.ac.rs (I.D.); ana.ninic@pharmacy.bg.ac.rs (A.N.); jelena.vekic@pharmacy.bg.ac.rs (J.V.); taravorkapic@gmail.com (T.V.); sanja.erceg@pharmacy.bg.ac.rs (S.E.); tamara.gojkovic@pharmacy.bg.ac.rs (T.G.); 2 Faculty of Medicine, University of Belgrade, 11000 Belgrade, Serbia; ratko.tomasevic@med.bg.ac.rs; 3 Department of Gastroenterology and Hepatology, Clinic for Internal Medicine, Clinical Hospital Center Zemun, 11080 Belgrade, Serbia; 4 Department of Laboratory Diagnostics, Clinical Hospital Center Zemun, 11080 Belgrade, Serbia; mamicmilics8@gmail.com; 5 Clinical Department for Gastroenterology and Hepatology, University Medical Center Zvezdara, 11000 Belgrade, Serbia; dr.milosh.mitrovic@gmail.com

**Keywords:** MASLD, plasma fatty acids, n-6 PUFAs, n-3 PUFAs, linoleic acid

## Abstract

The increasing prevalence of metabolic dysfunction-associated steatotic liver disease (MASLD) underscores the need for better understanding of its complex pathogenesis. Lipid accumulation in hepatocytes is among principal mechanisms contributing to MASLD development. While routine lipid parameters are well studied, the profile of circulating fatty acids in MASLD patients remains less explored. This study aimed to assess relative proportions of individual fatty acids in plasma of MASLD patients and to explore their associations with other biochemical markers of MASLD. Ninety-one patients and 48 healthy individuals were enrolled. The relative proportions of fatty acids in plasma were determined using gas chromatography with FID detection. Proportions of total n-6 polyunsaturated fatty acids (PUFAs) and linoleic acid (LA) in plasma were lower in MASLD patients (*p* = 0.001 and *p* = 0.004, respectively), with no differences observed in n-3 PUFAs. Total plasma n-6 PUFAs correlated negatively with body mass index, hepatic steatosis indices, triglyceride concentration and coronary risk index. Decreased prevalence of n-6 PUFAs in plasma was independently associated with higher odds of MASLD (OR = 0.769; CI: 0.611–0.968; *p* = 0.025). Our findings indicate an altered circulatory fatty acid distribution in MASLD, characterized by a reduced amount of n-6 PUFAs, particularly LA, which may have significant implications for the prevention and treatment of MASLD.

## 1. Introduction

Metabolic dysfunction-associated steatotic liver disease (MASLD) is the most common liver disease in the modern world [1,2]. The prevalence of MASLD is rising alongside the global burden of obesity, since metabolic alterations related to the development of hepatic steatosis are typically driven by obesity. Also, there is an alarming trend of increased MASLD-associated mortality rate, with cancer and cardiovascular diseases being predominant causes of death in this population [2,3].

It has been reported that several pathophysiological mechanisms can contribute to the onset of MASLD, but lipotoxicity plays a principal role in the development of hepatocyte dysfunction. Accumulation of triglycerides (TG) and free fatty acids in hepatocytes triggers lipotoxic effects, leading to elevated oxidative stress, cellular dysfunction and apoptosis, ultimately causing liver fibrosis [4]. Although the contribution of dyslipidemia in the pathogenesis of MASLD is widely appreciated, only a limited number of studies have analyzed fatty acid composition in plasma of these patients, so reliable conclusions are still lacking in this field.

Fatty acids in circulation originate either from dietary intake or endogenous de novo synthesis and hydrolysis of TG. As components of complex lipids, they are essential for energy metabolism and maintaining the integrity of cell membranes and cell signaling [5]. The specific contribution of saturated and unsaturated fatty acids (SFAs and UFAs, respectively) to cardiometabolic risk is well known [6]. Likewise, the role of n-6 and n-3 polyunsaturated fatty acids (PUFAs) has been extensively explored in cardiometabolic diseases. However, a full consensus on the significance and range of their effects has still not been reached [7,8,9,10]. The physiological importance of PUFAs is mainly related to biological actions of the essential fatty acids, n-6 linoleic acid (18:2 (n-6), LA), n-3 α-linolenic acid (18:3 (n-3), ALA), and their metabolites: arachidonic acid (20:4 (n-6), AA), eicosapentaenoic acid (20:5 (n-3), EPA) and docosahexaenoic acid (22:6 (n-3), DHA) [11]. It is generally accepted that metabolic transformations of n-6 PUFAs lead to the production of pro-inflammatory mediators, while n-3 PUFAs are associated with beneficial cardiometabolic effects [12,13]. However, the impact of these specific PUFAs on the onset and progression of MASLD has not been sufficiently explored. Recent evidence suggests that increased dietary intake of both n-6 and n-3 PUFAs is inversely associated with the risk for MASLD [14]. Since dietary and lifestyle changes represent a first-line treatment for MASLD, understanding the circulatory fatty acid profile in patients with MASLD could significantly improve therapeutic strategies by targeting specific bioactive molecules.

The primary aim of this study was to analyze the plasma fatty acid profile in individuals with MASLD and investigate how specific fatty acids relate to traditional cardiometabolic risk factors. Additionally, the study sought to determine whether plasma fatty acids could be independently associated with the presence of MASLD. Insights into the role of fatty acids in MASLD pathogenesis may contribute to broader efforts in preventing and managing MASLD and related metabolic disorders.

## 2. Materials and Methods

### 2.1. Patients

This study included a cohort of 100 adult patients diagnosed with hepatic steatosis, as confirmed by ultrasound examination. The exclusion criteria included viral hepatitis, HIV infection, celiac disease, hereditary liver diseases, and the usage of potentially hepatotoxic medications. MASLD was defined according to the criteria of the Delphi consensus statement [15]. None of the patients declared regular alcohol intake higher than 140 g/week for women or 210 g/week for men; thus, the diagnosis of metabolic and alcohol-associated liver disease (MAASLD) was excluded. MASLD criteria were not met in 7 out of 100 patients; therefore, in the absence of other liver-associated pathological conditions, these cases were classified as cryptogenic steatotic liver disease. Complete laboratory data were not collected in 2 cases, so the final patient group consisted of 91 patients with MASLD. Diabetes was present in 54 MASLD cases and hypertension in 32 cases. The control group involved 48 volunteers with no signs of hepatic steatosis, according to the ultrasound examination. Exclusion criteria for the control group comprised the presence of diabetes, coronary heart disease, any other metabolism-associated disorder and use of lipid-lowering medications.

Patients were recruited between January 2020 and March 2023 at the University Medical Centers Zemun and Zvezdara, while the control group was recruited at the University of Belgrade—Faculty of Pharmacy. Questionnaires were administered to gather demographic and clinical information, including age, gender, body weight, height, waist circumference, hip circumference, systolic and diastolic blood pressure, presence of other diseases and current medications. Additionally, data on lifestyle habits, such as smoking status, alcohol consumption and dietary habits, were collected. Regarding nutritional habits, all participants can be allocated to the traditional Central European dietary pattern. Body mass index (BMI) was calculated using the formula: weight (kg)/height (m)^2^.

The participants were informed of the study’s objectives and gave written consent before participation. The study protocol received approvals from the Ethics Committees of the University of Belgrade—Faculty of Pharmacy, as well as from the University Medical Centers Zemun and Zvezdara.

### 2.2. Laboratory Methods

Fasting blood samples were taken for separation of serum and plasma with K_2_EDTA anticoagulant. Plasma and serum were separated by centrifugation at 1500 rcf for 10 min. Routine laboratory analyses were performed promptly. Total protein, glucose, uric acid, total cholesterol (TC), triglycerides (TG), and HDL-cholesterol (HDL-C) were measured in serum using routine spectrophotometric methods on the DxC 700 AU and DxC 480 AU automated analyzers from Beckman Coulter (Brea, CA, USA). In addition, C-reactive protein (CRP) levels were quantified in serum by the immunoturbidimetric method. LDL-cholesterol was calculated using the Friedewald formula. Coronary risk index was calculated as TC/HDL-C ratio and the atherosclerosis index as LDL-C/HDL-C ratio. The degree of hepatic steatosis was assessed by hepatic steatosis indices. Hepatic steatosis index (HSI) values were calculated using the formula: 8 × [alanine aminotransferase (ALT)/aspartate aminotransferase (AST)] + body mass index (BMI) + 2 (if diabetic) + 2 (if female) [16]. The triglyceride and glucose index (TyG) was calculated using the formula: ln[TG (mg/dL) × Glucose (mg/dL)/2] [17]. Both the plasma and the rest of the serum were divided into several aliquots, which were stored at −80 °C until further analyzed. The samples were thawed and mixed just before analysis.

### 2.3. Analysis of Total Plasma Fatty Acid Composition

Fatty acids’ composition was analyzed in plasma samples after direct in situ extraction and trans-esterification according to the method described by Glaser et al. [18]. Briefly, 300 µL of plasma and 1.5 mL of 3M HCl in methanol were combined in closed glass tubes to obtain fatty acid methyl esters (FAMEs). Plasma with solvents for extraction and methylation were vortexed for 30 s and then heated in a water bath at 85 °C for 45 min. After cooling to room temperature, 0.5 mL of hexane (Sigma Aldrich, Saint Louis, MO, USA) was added and vortexed for 30 s to extract FAMEs. Centrifugation at 3000 rpm for 5 min was performed to separate the hexane layer containing FAMEs. An aliquot of the upper hexane phase was transferred into 2 mL vials, and FAMEs were analyzed using gas chromatography on an Agilent 7890 instrument with flame ionization detection and capillary column (CP-Sil88; 100 m × 0.25 mm, 0.2 μm film thickness; SUPELCO, Bellefonte, PA, USA).

GC analysis was performed under the following conditions: 1 μL of FAMEs mixture was injected in split mode 20:1; the injector temperature was set to 250 °C; the injector split flow to 20 mL/min, the pressure to 31,623 psi. The oven temperature program started at 80 °C and was raised by 4 °C/min up to 220 °C (hold time 5 min), then by 4 °C/min up to 240 °C, and then was held at 240 °C for 10 min. The carrier gas (He) flow rate was set to 1.0 mL/min and the makeup gas nitrogen flow was set to 25 mL/min. The FID detector operated at a temperature of 270 °C, and the run time was 55 min. Chromatographic peaks were identified by comparing their retention times with a standard FAME mix (Supelco FAME Mix, Bellefonte, PA, USA).

The percentage of individual fatty acids was calculated as the ratio between each individual chromatographic peak area and the sum of all peak areas. The results were presented as the percentage of each fatty acid. Relative proportions in plasma were estimated, and comparison analysis was performed for the following fatty acids: palmitic acid (16:0, PA), stearic acid (18:0, SA), oleic acid (18:1 (n-9), OA), linoleic acid (18:2 (n-6), LA), arachidonic acid (20:4 (n-6), AA), eicosapentaenoic acid (20:5 (n-3), EPA), docosapentaenoic acid (22:5 (n-3), DPA) and docosahexaenoic acid (22:6 (n-3), DHA), given that these specific fatty acids were present in the amounts that exceeded the limit of quantification (signal-to-noise ratio ≥ 10) in each analyzed sample. Total SFA and UFA percentages were calculated by summing relative proportions of individual SFAs and UFAs in the total fatty acid profile, while total n-6 PUFAs and n-3 PUFAs were estimated as the sums of relative proportions of separate n-6 and n-3 PUFAs. The n-6/n-3 PUFA ratio was determined by dividing the sums of total n-6 PUFAs and total n-3 PUFAs. Likewise, the AA/EPA ratio and AA/LA ratio were determined by dividing the relative proportions of these specific fatty acids.

### 2.4. Statistical Analysis

Normality of data distribution was tested by the Kolmogorov–Smirnov test. Normally distributed variables were presented as mean ± standard deviations, and group differences were examined by the Student *t*-test, whereas asymmetrically distributed variables were given as median (interquartile range) and analyzed by the Mann–Whitney U-test. Categorical data are presented as percentages and compared by the Chi-square test. Correlation analysis was performed by using Spearman’s correlation coefficient. Univariate and multivariate logistic regression analysis was used to assess independent associations of specific factors with the presence of MASLD. Differences were considered significant if *p* < 0.05. All statistical analyses were executed by using the statistical package PASW Statistics 21.0 (IBM, Armonk, NY, USA).

## 3. Results

The general anthropometric characteristics of the study participants are given in Table 1. There were no significant differences in age, gender distribution and lifestyle habits among MASLD patients and the control group. Expectedly, BMI, waist-to-hip ratio and HSI were higher in the MASLD group. Similarly, this group had higher concentrations of glucose, uric acid and CRP. Regarding serum lipid parameters, there were no differences in TC and LDL-C levels, but HDL-C concentrations were significantly lower, while TG concentrations were higher in subjects with MASLD compared to controls. Coronary risk index and atherosclerosis index were also higher in the MASLD group.

Table 2 represents the analysis of circulatory fatty acid profiles in MASLD patients and control subjects. The obtained results showed that the relative proportions of saturated PA were significantly higher in patients, while the percentage of polyunsaturated n-6 LA was higher in the control group. There were no significant differences in proportions of AA, EPA, DPA and DHA among the groups. The contribution of SFA in total fatty acid distribution was higher, and the contribution of UFAs was lower in MASLD patients than in the control group. Total n-6 PUFAs were more prevalent in controls. Although there were no significant differences in the following parameters, a trend of lower n-6/n-3 PUFAs and higher AA/EPA and AA/LA ratios was observed in subjects with MASLD.

Spearman’s correlation analysis in a total analyzed cohort (Table 3) revealed significant positive associations of PA with BMI, waist-to-hip ratio, HSI, TyG index, levels of glucose and TG, as well as with coronary risk and atherosclerosis indices. In contrast, the percentage of PA negatively correlated with HDL-C. The relative proportion of OA was in negative correlation with TC, LDL-C and the atherosclerosis index. Polyunsaturated LA negatively correlated with BMI, waist-to-hip ratio, HSI, TyG index, as well as with glucose, uric acid, CRP and TG levels and TC/HDL-C ratio, whilst positively with HDL-C concentration. The relative proportion of AA was in positive association with uric acid concentrations. The total proportion of SFAs positively correlated with BMI, waist-to-hip ratio, HSI, TyG index, uric acid, TG levels and coronary risk index, while negatively with HDL-C concentrations. The percentage of total UFAs correlated with the same parameters as SFAs, but in the opposite directions. The relative proportion of n-6 PUFAs was in negative correlation with BMI, HSI, TyG index, glucose uric acid and TG levels, whilst in positive correlation with concentrations of TC, LDL-C and HDL-C. Finally, the AA/LA ratio had a significant positive correlation with the waist-to-hip ratio, TyG index and levels of uric acid and TG.

The association of specific plasma fatty acids with probability of MASLD occurrence was tested by using univariate and multivariate logistic regression analysis (Table 4 and Table 5, respectively). According to the univariate analysis, the proportions of PA and total SFAs were found to be in positive associations with higher odds of presence of MASLD. On the other hand, relative proportions of LA, total UFAs, n-6 PUFAs and n-6/n-3 ratio were revealed as negatively associated with probability of MASLD development. However, when each of the selected markers was included in a multivariate model consisting of traditional risk factors for MASLD, only a relative proportion of n-6 PUFAs and n-6/n-3 ratio retained their significance as independent indicators of higher probability of the presence of MASLD.

## 4. Discussion

In this study, we have demonstrated altered circulatory fatty acid profiles in patients with MASLD when compared to healthy individuals, whereas main differences originated from a decreased amount of n-6 PUFAs, specifically LA, in MASLD. Moreover, our results suggest significant negative correlations of n-6 PUFAs with obesity and inflammatory markers, as well as independent association of these fatty acids with the presence of MASLD.

Analysis of anthropometric and biochemical markers indicated typical changes for MASLD, i.e., increased obesity markers, hepatic steatosis indices and parameters of glycemia, dyslipidemia and inflammation (Table 1). The pathogenesis of MASLD is frequently explained by the “multiple hit” hypothesis, which involves the simultaneous presence of TG accumulation in the liver, lipotoxicity, insulin resistance, oxidative stress and excessive inflammatory response [19,20]. Novel research repeatedly points towards common molecular mechanisms, which bring together these different pathways and shed light on the role of peroxisome proliferator-activated receptors (PPARs) in counteracting these processes [21,22]. Namely, each of the PPARs—PPARα, PPARβ/δ and PPARγ—is involved in maintaining metabolic homeostasis, which makes them eligible candidates for the treatment of MASLD. PUFAs can activate PPARs [23], thus contributing to their favorable effects. In the current study, the amount of total SFAs was higher, while the levels of total UFAs and total n-6 PUFAs were lower in patients with MASLD (Table 2), suggesting the lack of PPAR ligands. Likewise, it is well known that PUFAs can inhibit lipogenesis by suppressing proteolysis of its key modulators—sterol regulatory element-binding proteins [24]. Therefore, a decreased amount of total UFAs and certain PUFAs can presumably lead to the altered synthesis of various lipid moieties in MASLD. Although our results demonstrated that the levels of TC and LDL-C were comparable among groups, HDL-C was significantly lower, while TG concentrations were higher in MASLD patients (Table 1). Such findings are typical for MASLD and align with the lack of UFAs. Recent Mendelian randomization analyses have revealed negative associations of HDL-C and positive link of TG with MASLD [25,26]. In addition, altered structural and functional properties of HDL have also been found in MASLD patients [27,28]. These results underline the well-known importance of HDL in providing cardiometabolic protection [29]. Interestingly, a study conducted by Mocciaro et al. [30] demonstrated a depletion of PUFAs in total serum and HDL fraction of MASLD patients, indicating that altered functionality of HDL in MASLD might at least partly be due to a lack of PUFA-enriched phospholipids in its structure. We previously reported changes in HDL and LDL subclasses distribution in diabetes patients treated with a PPARγ agonist [31], thereby supporting the presumption that PUFAs could affect lipoproteins’ structure and functionality by acting as PPAR ligands. It should also be noted that in the current study, total SFAs and total UFAs reciprocally correlated with hepatic steatosis indices, as well as with cardiovascular risk indices (Table 3), hence implying the involvement of specific fatty acids in the development of both MASLD and its cardiovascular co-morbidities.

An intriguing finding of this study is the observed decreased amount of total n-6 PUFAs and LA, but not n-3 PUFAs in subjects with MASLD (Table 2). This is a surprising result, since it has been widely accepted that n-3 PUFAs are associated with favorable metabolic effects and decreased risk for MASLD [32,33]. Neither n-3 PUFA content nor n-6/n-3 PUFA ratio differed between patients and controls (Table 2). Of note, recent results from a large cohort have demonstrated an inverse association of n-6 PUFAs and specifically LA with the odds of hepatic steatosis [34]. These results are in line with our findings of an independent association of decreased plasmatic n-6 PUFAs with MASLD (Table 5). It should be mentioned that higher BMI and prevalence of obesity in MASLD patients might affect the plasma fatty acid profile and its relationship with the disease development. However, the results of multivariate logistic regression analysis (Table 5) imply that obesity does not lay behind the link between decreased n-6 PUFAs and higher odds of MASLD. Although n-6 PUFAs and their metabolites are generally considered as pro-inflammatory mediators [35], available evidence indicates that both LA and AA might also be linked to anti-inflammatory effects. Namely, it has been reported that increased dietary intake of both LA and AA in appropriate amounts does not enhance inflammatory response but can even be associated with reduced inflammation [36]. In addition, an inverse relation has been shown between dietary LA intake and the risk of liver fibrosis [37]. In our study, decreased LA in plasma was related to higher odds of MASLD, although this association did not remain after the adjustment for traditional MASLD risk factors. Nevertheless, LA is the most prevalent fatty acid in the entire PUFA profile, so its contribution to the observed independent association of total n-6 PUFAs with MASLD (Table 5) should not be neglected.

It is important to note that, even if we demonstrated a decreased amount of LA in the plasma of MASLD patients, there were no differences in the percentage of its principal metabolite AA and AA/LA ratios among the groups. Although a relatively small sample size might be responsible for such a lack of differences, another possibility should also be considered. Namely, it is possible that a relative excess of LA in healthy individuals could be metabolized by alternative routes. Recently, much attention has been paid to conjugated LA metabolites, which are produced in humans by gut microbiota [38] and exert many beneficial effects, including anti-obesogenic, anti-inflammatory, anti-oxidative, anti-neoplastic and anti-atherosclerotic roles [39,40]. In line with these findings, our results of consistent negative correlation between plasma LA amount and markers of obesity, dyslipidemia, inflammation and atherosclerosis risk (Table 3) support the presumption that these associations can at least partly be attributed to LA-conjugated metabolites. Moreover, it has been shown that LA metabolites produced by gut microbiota can ameliorate hepatic steatosis, inflammation and fibrosis [41], which is in line with our findings of negative associations between LA and hepatic steatosis indices. Such recent evidence confers a novel insight into the role of n-6 PUFAs in MASLD and warrants further investigations.

To the best of our knowledge, a limited number of studies analyzed the independent contribution of specific fatty acids to the risk for the development of MASLD. A recent large-scale study, based on data provided by NHANES, has demonstrated negative associations between dietary LA intake and the risk of liver fibrosis [37]. Our study shows that lower relative proportions of LA and total n-6 PUFAs in plasma are associated with higher odds of the presence of MASLD, thus confirming and extending such previous findings. LA is the most abundant PUFA in a typical Western diet, which implies a broad spectrum of possibilities for dietary interventions in order to reduce the risk of MASLD. However, further large-scale studies are needed to evaluate these preliminary findings of the beneficial effects of n-6 PUFAs, particularly LA in counteracting MASLD development.

Several constraints should be considered. First, a cross-sectional design of the research prevented the exploration of causal relationships between the abundance of specific fatty acids in plasma and MASLD development. The observed associations of lower plasma n-6 PUFAs with higher odds of MASLD occurrence should be examined by prospective studies, to reveal the possible prognostic capacity of decreased circulatory n-6 PUFAs for the onset of MASLD. Second, we did not analyze further metabolic transformations of fatty acids, so potential differences between patients and controls in microbiota-derived metabolites and their roles in MASLD development should be explored in future studies. Next, although there were no statistically significant differences in age and gender between the examined groups, there was a trend of older age and higher prevalence of men in the MASLD group. However, the multivariate regression model included age and gender as covariates to minimize such differences. Further studies with a larger sample size are necessary to explore the significance of the observed findings in both genders and various age groups. In conclusion, the results presented herein demonstrate that the fatty acid profile in MASLD patients is characterized by an elevated presence of SFAs and a reduction in n-6 PUFAs, particularly LA. Furthermore, significant inverse correlations were observed between n-6 PUFAs and markers of hepatic steatosis and cardiometabolic risk. Notably, reduced plasma levels of n-6 PUFAs were identified as being independently associated with higher odds of MASLD. These findings extend the existing knowledge on the role of fatty acids in metabolic perturbations that lead to MASLD development and lay the groundwork for further research aimed at a thorough understanding of MASLD pathogenesis.

## Figures and Tables

**Table 1:** Comparison of general clinical and biochemical characteristics among the studied groups.

Parameter	MASLD (N = 91)	Control Group (N = 48)	*p*
Age (years)	53.70 ± 11.62	49.35 ± 14.11	0.054
Gender, male (%)	44.0	27.1	0.051
Smoking, yes (%)	34.1	29.2	0.557
Occasional alcohol intake, no (%)	67.0	68.1	0.373
Physical activity, yes (%)	49.4	54.4	0.589
BMI, kg/m^2^	29.62 ± 4.18	24.59 ± 2.98	<0.001
Waist-to-hip ratio	0.93 ± 0.09	0.83 ± 0.10	<0.001
Systolic pressure (mm Hg)	126.9 ± 13.2	121.9 ± 14.9	0.066
Diastolic pressure (mm Hg)	79.4 ± 10.6	76.9 ± 10.3	0.231
HSI *	41.44 (37.23–45.48)	33.28 (31.90–35.12)	<0.001
TyG index *	8.90 (8.58–9.34)	8.25 (7.89–8.71)	<0.001
Total protein (g/L)	71.21 ± 5.95	71.63 ± 3.98	0.663
Glucose (mmol/L)	6.60 ± 2.30	5.11 ± 0.47	<0.001
Uric acid (μmol/L) *	322.0 (271.0–379.5)	261.0 (203.5–301.0)	<0.001
CRP (mg/L) *	2.60 (1.50–5.10)	0.94 (0.50–2.55)	<0.001
TC (mmol/L)	5.38 ± 1.09	5.56 ± 1.38	0.396
LDL-C (mmol/L)	3.23 ± 0.93	3.40 ± 1.18	0.353
HDL-C (mmol/L)	1.39 ± 0.47	1.66 ± 0.35	<0.001
TG (mmol/L) *	1.56 (1.21–2.10)	0.92 (0.74–1.46)	<0.001
Coronary risk index *	4.12 (3.33–4.84)	3.39 (2.83–3.90)	<0.001
Atherosclerosis index *	2.56 (1.77–3.04)	2.01 (1.62–2.66)	0.012

Data are presented as mean ± standard deviation and compared by the Student *t*-test. Categorical data are presented as relative frequencies (%) and compared by the Chi-square test. * Data are presented as median (interquartile range) and compared by the Mann–Whitney U test.

**Table 2:** Comparison of plasma fatty acid profiles between MASLD patients and control group.

Parameter	MASLD (N = 91)	Control Group (N = 48)	*p*
16:0, PA (%) *	29.67 ± 1.74	28.69 ± 1.80	0.002
18:0, SA (%) *	12.12 ± 1.30	11.90 ± 1.12	0.312
18:1 (n-9), OA (%) *	14.13 ± 1.94	13.86 ± 2.19	0.449
18:2 (n-6), LA (%) *	26.00 ± 3.58	27.84 ± 3.44	0.004
20:4 (n-6), AA (%)	13.70 (11.90–16.30)	14.30 (12.30–16.10)	0.861
20:5 (n-3), EPA (%)	0.25 (0.15–0.37)	0.26 (0.20–0.42)	0.277
22:5 (n-3), DPA (%)	0.47 (0.32–0.63)	0.46 (0.29–0.68)	0.770
22:6 (n-3), DHA (%)	3.02 (2.49–3.75)	3.05 (2.31–3.44)	0.273
Total SFAs (%)	42.10 (40.30–43.00)	40.70 (39.25–41.85)	<0.001
Total UFAs (%)	57.92 (56.95–59.67)	59.22 (58.13–60.62)	0.001
Total n-6 PUFAs (%)	39.90 (38.00–42.00)	41.40 (40.00–43.15)	0.001
Total n-3 PUFAs (%)	3.57 (3.22–4.75)	3.73 (2.98–4.62)	0.414
n-6/n-3 PUFA ratio	10.79 (8.36–13.11)	11.11 (9.12–14.17)	0.210
AA/EPA ratio	56.58 (36.55–86.43)	50.00 (29.77–74.57)	0.167
AA/LA ratio	0.53 (0.42–0.67)	0.51 (0.42–0.60)	0.241

Data are presented as median (interquartile range) and compared by the Mann–Whitney U test. * Data are presented as mean ± standard deviation and compared by the Student *t*-test. 16:0, PA—palmitic acid; 18:0, SA—stearic acid; 18:1 (n-9), OA—oleic acid; 18:2 (n-6), LA—linoleic acid; 20:4 (n-6), AA—arachidonic acid; 20:5 (n-3), EPA—eicosapentaenoic acid; 22:5 (n-3), DPA—docosapentaenoic acid; 22:6 (n-3), DHA—docosahexaenoic acid; SFAs—saturated fatty acids; UFAs—unsaturated fatty acids; PUFAs—polyunsaturated fatty acids.

**Table 3:** Significant correlations of components of fatty acid profiles with other examined parameters.

Parameter	16:0, PA (%)	18:1 (n-9), OA (%)	18:2 (n-6), LA (%)	20:4 (n-6), AA (%)	Total SFAs (%)	Total UFAs (%)	Total n-6 PUFAs (%)	AA/LA Ratio
BMI (kg/m^2^)	0.264 **		−0.250 **		0.321 ***	−0.329 ***	−0.271 **	
Waist-to-hip ratio	0.323 **		−0.321 **		0.309 **	−0.297 **		0.225 *
HSI	0.306 ***		−0.302 ***		0.375 ***	−0.374 ***	−0.305 ***	
TyG index	0.389 ***		−0.425 ***		0.380 ***	−0.384 ***	−0.368 ***	0.266 **
Glucose (mmol/L)	0.249 **		−0.200 *				−0.171 *	
Uric acid (μmol/L)			−0.233 **	0.189 *	0.187 *	−0.189 *		0.210 *
CRP (mg/L)			−0.177 *					
TC (mmol/L)		−0.363 ***					0.169 *	
HDL-C (mmol/L)	−0.384 ***		0.283 **		−0.329 ***	0.318 ***	0.333 ***	
LDL-C (mmol/L)		−0.382 ***					0.189 *	
TG (mmol/L)	0.368 ***		−0.414 ***		0.396 ***	−0.408 ***	−0.360 ***	0.259 **
Coronary risk index	0.311 ***		−0.192 *		0.282 **	−0.276 **		
Atherosclerosis index	0.175 *	−0.216 **						

Values represent Spearman’s correlation coefficients. * *p* < 0.05; ** *p* < 0.01; *** *p* < 0.001. 16:0, PA—palmitic acid; 18:1 (n-9), OA—oleic acid; 18:2 (n-6), LA—linoleic acid; 20:4 (n-6), AA—arachidonic acid; SFAs—saturated fatty acids; UFAs—unsaturated fatty acids; PUFAs—polyunsaturated fatty acids.

**Table 4:** Univariate logistic regression analysis of associations of fatty acids with MASLD.

Parameter	OR	95% CI	*p*
16:0, PA (%)	1.366	1.108–1.683	0.003
18:2 (n-6), LA (%)	0.860	0.772–0.957	0.006
Total SFAs (%)	1.438	1.162–1.779	0.001
Total UFAs (%)	0.747	0.611–0.913	0.004
Total n-6 PUFAs (%)	0.793	0.686–0.917	0.002
n-6/n-3 PUFA ratio	0.914	0.838–0.997	0.043

Data are presented as odds ratio (OR) and 95% confidence intervals. 16:0, PA—palmitic acid; 18:2 (n-6), LA—linoleic acid; SFAs—saturated fatty acids; UFAs—unsaturated fatty acids; PUFAs—polyunsaturated fatty acids.

**Table 5:** Multivariate logistic regression analysis of association of fatty acids with MASLD.

Parameter	OR	95% CI	*p*
16:0, PA (%)	1.187	0.871–1.618	0.278
18:2 (n-6), LA (%)	0.875	0.735–1.042	0.135
Total SFA (%)	1.258	0.949–1.669	0.111
Total UFA (%)	0.821	0.619–1.088	0.170
Total n-6 PUFAs (%)	0.769	0.611–0.968	0.025
n-6/n-3 PUFA ratio	0.811	0.709–0.928	0.002

Data are presented as odds ratio (OR) and 95% confidence intervals. Variables included in the model: age, gender (0-female, 1-male), smoking (0-no, 1-yes), physical activity (0-no, 1-yes), alcohol consumption (0-no, 1-yes), BMI and concentrations of glucose and HDL-C. 16:0, PA—palmitic acid; 18:2 (n-6), LA—linoleic acid; SFAs—saturated fatty acids; UFAs—unsaturated fatty acids; PUFAs—polyunsaturated fatty acids.

## Data Availability

The data presented in this study are available upon request from the corresponding author. The data are not publicly available due to privacy and ethical considerations.
